# Community perspectives on AI/ML and health equity: AIM-AHEAD nationwide stakeholder listening sessions

**DOI:** 10.1371/journal.pdig.0000288

**Published:** 2023-06-30

**Authors:** Jamboor K. Vishwanatha, Allison Christian, Usha Sambamoorthi, Erika L. Thompson, Katie Stinson, Toufeeq Ahmed Syed

**Affiliations:** 1 Institute for Health Disparities, University of North Texas Health Science Center, Fort Worth, Texas, United States of America; 2 College of Pharmacy, University of North Texas Health Science Center, Fort Worth, Texas, United States of America; 3 Department of Biostatistics and Epidemiology, School of Public Health, University of North Texas Health Science Center, Fort Worth, Texas, United States of America; 4 Department of Biomedical Informatics, Vanderbilt University Medical Center, Nashville, Tennessee, United States of America; The University of Melbourne, AUSTRALIA

## Abstract

Artificial intelligence and machine learning (AI/ML) tools have the potential to improve health equity. However, many historically underrepresented communities have not been engaged in AI/ML training, research, and infrastructure development. Therefore, AIM-AHEAD (Artificial Intelligence/Machine Learning Consortium to Advance Health Equity and Researcher Diversity) seeks to increase participation and engagement of researchers and communities through mutually beneficial partnerships. The purpose of this paper is to summarize feedback from listening sessions conducted by the AIM-AHEAD Coordinating Center in February 2022, titled the “AIM-AHEAD Community Building Convention (ACBC).” A total of six listening sessions were held over three days. A total of 977 people registered with AIM-AHEAD to attend ACBC and 557 individuals attended the listening sessions across stakeholder groups. Facilitators led the conversation based on a series of guiding questions, and responses were captured through voice and chat via the Slido platform. A professional third-party provider transcribed the audio. Qualitative analysis included data from transcripts and chat logs. Thematic analysis was then used to identify common and unique themes across all transcripts. Six main themes arose from the sessions. Attendees felt that storytelling would be a powerful tool in communicating the impact of AI/ML in promoting health equity, trust building is vital and can be fostered through existing trusted relationships, and diverse communities should be involved every step of the way. Attendees shared a wealth of information that will guide AIM-AHEAD’s future activities. The sessions highlighted the need for researchers to translate AI/ML concepts into vignettes that are digestible to the larger public, the importance of diversity, and how open-science platforms can be used to encourage multi-disciplinary collaboration. While the sessions confirmed some of the existing barriers in applying AI/ML for health equity, they also offered new insights that were captured in the six themes.

## Author summary

Artificial intelligence and machine learning (AI/ML) have gained significant traction in the field of healthcare over the past several years. Innovative clinical applications are at the forefront of this “next frontier;” however, AI/ML can also be used to push the envelope for health equity by providing these tools to stakeholders at the grassroots level. We held nation-wide listening sessions with stakeholders from diverse organizations and institutions who are interested in using AI/ML to address health disparities, in order to shed light on the unique barriers these groups face. We were able to gather insight on how AI/ML tools could be improved, opportunities to increase diversity in data science, and using trusted community networks to ensure sustainability. This manuscript offers an approach to improving AI/ML data, infrastructure, and training that places community voices at the forefront in order to empower stakeholders to effect change.

## Introduction

Artificial intelligence (AI) involves computer science approaches to designing applications that can carry out intelligent tasks [1,2]. Machine learning (ML) is a subset of AI where computer algorithms can make predictions for complex tasks based on learning from input examples [1,3]. There are different ways in which the machine can learn from the data, including supervised learning, unsupervised learning, and reinforcement learning [1,4]. AI/ML is increasingly prevalent in tools that we engage with daily, from shopping recommendations to aviation technology. The biomedical field could also benefit from augmenting human intelligence with cutting-edge technologies [2,4,5,6]. The collection of healthcare data, such as electronic health records (EHR), could act as a touchpoint for applying AI/ML tools to improve quality of care [7]. AI/ML applications in healthcare could involve supporting clinical decision making, identifying patterns in imaging, improving workflow efficiency, and reducing errors [1,6]. These exciting applications for healthcare professionals, patients, researchers, and communities are valuable tools that can be added to the clinician’s toolbelt. For example, researchers suggest that AI algorithms can perform “on par or better than humans in various tasks” [8, pg. 25]. With the addition of deep learning (DL), which is a type of ML, the computer can identify more complex patterns through artificial neural networks. These neural networks work in a way that mimics neurons in the human brain, but recognize patterns exponentially quicker and can handle big data [8]. “Big data” is a term used to describe multiple sources of complex health data. These data science methods therefore are extremely valuable in analyzing the large amount of EHR data, community data, public health surveillance records, and Social Determinants of Health (SDoH) data that are being collected [9].

SDoH are social factors such as built environment, access to healthcare, education, and distribution of resources [10,11]. SDoH influence health directly, and there are structures and systems that disadvantage certain groups of people in SDoH domains which can lead to health disparities. Health disparities are differences in health and well-being among populations or groups of people, such as burden of disease or discrimination [10,12]. By contrast, health equity is the idea that everyone should have the ability to achieve optimal health and a good quality of life, and being equitable goes beyond just the absence of disparities. Core principles of health equity are taking deeper root in nationwide objectives to improve health with a focus on educating communities to improve health literacy, addressing historical injustices, and ensuring fairness in access to opportunity [10]. AI can either deepen health disparities or improve health equity, depending on how the tools are developed and applied. Examples of the positive potential are mentioned above, while an example of the negative potential can be seen with the widespread concern about racial bias in AI algorithms as a result of incomplete conceptualizations of data [9]. Other forms of bias in algorithms may include omitted variable bias, sampling bias, ascertainment bias, and measurement error [9]. Additionally, when thinking about the AI/ML data pipeline, it is not necessarily that the algorithms are being intentionally created with bias, but that the data feeding into the algorithms is inherently biased due to a lack of data from underrepresented groups, or data absenteeism, and an overrepresentation of data from more populous groups [9,13]. Therefore, it is important that AI initiatives keep an eye toward health equity and advocate for system level changes to better govern implementation of data science approaches [11]

Algorithms have the potential to significantly impact patients and communities, and it is vital that equity is a top priority [5]. ML approaches to equity can be described through the concept of “fairness” [9]. There are formal methods to applying fairness in AI/ML development such as anti-classification, classification parity, and calibration. These approaches allow for protection of variables to impact predictions, while examining accuracy and true risk prediction across groups [9]. Widespread use of formal definitions and applications to the notions of fairness and equity are needed to avoid harming those impacted by health disparities. One way AI/ML can contribute to the perpetuation of health disparities and inequities is through lack of diversity of both researchers and data [7,14]. For example, the 2021 Artificial Intelligence Index Report prepared by Zhang and colleagues discusses how the percentage of AI and computer science PhD graduates has been dominated by men, as well as White (45.6%) and Asian (22.4%) individuals. By contrast, less than 18% of AI PhD graduates in the United States identified as female as of 2019; only 3.2% were Hispanic and 2.4% were Black/African American [15]. Research teams engaged in AI/ML should be diverse in terms of demographics, skills, methods, disciplines, ideologies, and organization/institution affiliation [16]. This contributes to a diverse way of thinking about AI/ML solutions, and diverse lived experiences may lend themselves to creative health equity innovations. Moreover, the data that are used to train AI/ML algorithms should be diverse [16]. Several publicly available datasets currently do not represent racial/ethnic minorities, sexual/gender minorities, and other historically underrepresented groups. On the other hand, private data may be more diverse but are not widely accessible or generalizable to the larger population [16]. Many of the algorithms that have been created do not include individuals who represent an accurate subset of the United States population as a result. In order to ensure fairness, it is not enough to just design AI/ML tools that avoid harming one group, but rather the tools should be designed to encompass the unique needs and characteristics of all groups. These tools should also look for innovative solutions to complex problems where a focused effort is needed [3]. Viewing these problems from the perspective of equity and fairness should be the priority of both AI/ML developers and end-users. As such, the consortium model lends itself to bringing together different groups in order to tackle these issues holistically.

Instead of continuing to use algorithms that are non-transparent and were built with data lacking diversity, a proactive approach should be taken to develop models that promote health equity [3,14]. To incorporate equity and justice frameworks into the application of data analysis that can reflect relationships between SDoH and health, the six V’s of big data can be used [9]. These considerations include how broad the data are (volume), time of data collection (velocity), type of data (variety), trustworthiness (veracity), the potential impact (value), and obligation to equity to avoid perpetuating bias (virtuosity) [6]. Yet even with good algorithms, it may be difficult for organizations, institutions, and communities to implement AI/ML technologies due to cost, lack of infrastructure, and training. Historically underrepresented communities, which also face disproportionate health disparities, need to be at the table to add their rich insight to applying AI/ML to biomedical research questions [6]. Stakeholders must be included in every step of the process of creating AI/ML models and tools, from data collection to deployment [17]. In doing so, AI developers can collaborate with users to gain more insight into their lived experiences, historical/cultural context, and needs. An iterative co-development process between stakeholders, end-users, technical experts, and researchers can be key in developing AI/ML solutions to health disparity questions [17]. Although this can be more resource intensive for data scientists and researchers developing algorithms on the front-end, the opportunity to find innovative ways to dismantle health inequities would likely save on downstream healthcare costs once implemented [11]. Nevertheless, engaging stakeholders in AI/ML development has been a challenge thus far [18]. To ensure that these technologies will be adopted, in both clinical and community health settings, there has to be widespread approval from payers, clinicians, and regulators. In order to move AI/ML technologies into healthcare practice, efforts that focus on training and participatory research will be critical [18].

Furthermore, there needs to be an increased emphasis on using ethical frameworks for AI/ML to promote health equity and justice throughout the AI lifecycle [19,20]. Training is important to increase the general public’s understanding of how these models work and demystify the “black box” nature of algorithms [21,22] Particularly in healthcare, it is imperative that AI/ML is transparent. Transparency should be achieved through sets of measures applied to algorithm development, practice, and outcome predictions [21]. Stakeholders across disciplines must hold each other accountable in balancing how public and private sectors communicate about AI, document processes, manage and govern data, and develop shared meaning around algorithmic decision making. Furthermore, legal frameworks can assist in addressing AI/ML transparency at internal and external levels [21]. The National Institutes of Health (NIH) is “committed to leveraging the potential of AI/ML to accelerate the pace of biomedical innovation, while prioritizing and addressing health disparities and inequities” [7, p. 3]. In order to improve health equity, a transdisciplinary approach is needed and building strong partnerships with diverse stakeholders is vital to support data collection, curation, and use for AI/ML technology [7].

## Background

To advance health equity and researcher diversity in AI/ML, a consortium model was proposed by NIH with four key areas: partnership, research, infrastructure, and data science training. The consortium model is gaining momentum in the biomedical research community given the ability for stakeholders to share resources, collaborate on a shared vision, and build strong partnerships [23]. The AIM-AHEAD Coordinating Center (A-CC) was then developed, and includes institutions and organizations that have a core mission to serve minorities and other underrepresented or underserved groups impacted by health disparities [7]. The four key areas previously mentioned led to development of four cores and eight hubs that make up the

A-CC. The four cores include the Leadership/Administration Core (LAC), Data Science Training Core (DSTC), Data and Research Core (DRC), and Infrastructure Core (IC). Within the LAC, there are six hubs separated by region: North & Midwest Hub, West Hub, South Central Hub, Southeast Hub Meharry, Southeast Hub Morehouse, and Northeast Hub. The other two hubs within the LAC serve as overarching support: Central Hub and Communications & Dissemination Hub. The AIM-AHEAD Consortium consists of the A-CC (cores/hubs), NIH, partners, and stakeholders.

One goal of the A-CC includes gathering input from key stakeholders on their health disparities and AI/ML research priorities, training needs, and data and infrastructure needs in the near-, medium-, and long-terms. As learned through the NIH AIM-AHEAD Stakeholder Engagement Forum, developing a consortium on the topic of AI/ML with the goal of addressing health disparities requires thoughtful and early feedback from communities and stakeholders [24]. The thoughts, suggestions, and feedback from attendees help emphasize the need for aligning goals of the A-CC with needs of stakeholders. In February 2022, the A-CC held a series of nationwide listening sessions titled the AIM-AHEAD Community Building Convention (ACBC). The purpose of ACBC was to capture the AI/ML and health equity needs of the community for building and advancing the consortium, as well as to announce opportunities and potential resources. These sessions built upon the AIM-AHEAD Stakeholder Engagement Forum held by NIH in June 2021 prior to funding of the A-CC [25]. The event theme of ACBC was “Creating an Open Dialogue” as this was a great opportunity to continue the conversation with stakeholders. The convention included multiple virtual, discussion-based, moderated needs-gathering conversations. As learned from NIH, listening sessions were selected because they provide a space for the public to tell their stories, express their opinions, and share ideas [24].

## Methods

As an overview, we conducted nation-wide listening sessions that were developed to reach a large swath of persons from different fields (i.e., academic, industry, healthcare, community). Social media and other virtual networks were used for recruitment of participants to the listening sessions. Semi-structured questions were used to elicit participant perspectives about AI/ML and health equity, and feedback was collected via typed and verbal responses. Qualitative thematic analysis was used to assess de-identified transcripts from these sessions and identify key themes. The North Texas Regional Institutional Review Board determined this analysis of de-identified transcripts to be Not Human Subjects Research.

A Logistics Committee and Program/Theme Committee was developed with participation from all four A-CC cores. The Logistics Committee planned the event day/time, set up the meeting in HIPAA-compliant Zoom, developed plans for using the Slido platform (https://www.slido.com), hosted mock sessions for support staff, wrote scripts for moderators, created templates and checklists for notetakers/facilitators, etc. During the mock sessions a risk assessment was also performed for the moderators in the event that the conversation turned hostile, was hacked, or if they did not know how to respond to a participant. It was determined that in addition to plenty of A-CC members being in attendance during the sessions, Slack would also be used to communicate behind the scenes with the moderators and other A-CC support to assess any situations and to help the moderators in real time. Meanwhile, the Program/Theme Committee was charged with developing the content for the sessions, guiding questions, ACBC presentation slides, and pre- & post-surveys. The listening session categories were vetted by a team of experts within the A-CC. Sessions were developed with the mindset to ensure everyone felt free, safe, and comfortable sharing their thoughts, ideas, and suggestions. It was acknowledged that attendees’ voices, shared experiences, and insights will help AIM-AHEAD prioritize needs and resources in the AI/ML field. All levels of knowledge regarding AI/ML and healthy equity were welcomed to join.

## Materials

ACBC was held in February 2022, with two sessions per day over a three-day period.

The sessions were geared toward listening to the unique needs of various stakeholder groups, as determined by NIH during the creation of AIM-AHEAD and by consensus of the A-CC: (1) **Academic Institutions**—this session was held for those who work at all levels in educational institutions, such as (but not limited to): colleges, universities, minority serving institutions, etc.; (2) **Consortiums on AI/ML & Data Science**—this session was held for those in other consortiums, such as (but not limited to): consortiums on AI/ML, big data, data science, NIH-funded programs/organizations, etc.; (3) **Industry—**this session was held for those in industry, such as (but not limited to): business enterprises of all sizes, pharmaceutical & biotechnology industries, etc.; (4) **Healthcare—**this session was held for those in healthcare, such as (but not limited to): healthcare delivery systems, insurers/payers, regulatory bodies, healthcare providers, community-based health clinics, health-related associations/organizations, professional societies, local/regional/national healthcare advocates, local/state/federal health departments, etc.; (5) **Community-Based**—this session was held for those in community-based organizations, such as (but not limited to): advocacy/justice organizations, faith-based organizations, service-based organizations, non-profits, etc.; (6) **General Listening Session**—this session was held for individuals who felt that their affiliation did not align with one of the other categories, or if they wanted to know more about AI/ML and health equity. While it was thought that each of these groups would have unique perspectives regarding AI/ML and health equity, it was also assumed that there would be relatable themes present within each of the groups’ sessions. Participants self-identified which session they felt most closely aligned with their affiliation and/or interest.

The session began with a short introduction video featuring AIM-AHEAD’s Contact Multiple Principal Investigator, Dr. Jamboor Vishwanatha. Attendees were encouraged to engage verbally with moderators and other attendees during the sessions, and/or enter comments into the chat. Slido is a tool that was used for chatting, in which attendees could respond to particular topics and upvote comments they agreed with. Each session had three moderators, representing each of the four cores across the A-CC. There were also A-CC notetakers to take notes on major themes from the discussion. Lastly, the Central Hub from the LAC provided support staff to facilitate the Zoom sessions, provide technical support, and assist the moderators with monitoring the Slido chat. A pamphlet was sent out to registrants before the event in order to provide some background information on AIM-AHEAD and AI/ML, as well as introduce key terms that might be used during the sessions.

### Recruitment

Listening sessions were promoted through social media advertisements, emails, and snowball outreach procedures. To provide information about ACBC, a new page was created on the AIM-AHEAD.net website (https://aim-ahead.net/). This site served as a source of information as well as a registration portal for each of the listening sessions. The registration process for ACBC utilized a new software called SignUp, which was developed for the AIM-AHEAD Connect (https://connect.aim-ahead.net/) platform. When a user registered for a session, they were prompted to complete the registration fields and were then provided a link to add the event to their personal calendar. The registration fields included demographics, research interests, interest in AIM-AHEAD, if they will be attending other sessions, how they think AI/ML could support their interests/mission, what they were hoping to gain from joining AIM-AHEAD, any questions they would like to pose prior to the session, and if they would like to join the AIM-AHEAD Listserv. To lead people to the ACBC page, an alert banner was added to the top of every AIM-AHEAD.net page with information about ACBC.

The Marketing Committee launched recruitment for the ACBC in January 2022, by sending a Save the Date to all members of the A-CC. It was asked that members of the A-CC share this Save the Date with their networks, and then share their networks with the Marketing Committee in order to initiate AIM-AHEAD’s ‘Network of Networks’ for future outreach. The Save the Date was also shared via the AIM-AHEAD Listserv, on our social media channels (Twitter, Instagram, LinkedIn, and Facebook), and announced on AIM-AHEAD.net. Then the ACBC Marketing Kit was released to the A-CC containing a Press Release, an informational flyer, social media post, and messaging to accompany any emails or social posts. The flyer and social media posts were also shared with NIH, the National Research Mentoring Network (NRMN), the Community Engagement Alliance (CEAL) Against COVID-19 Disparities, Texas Center for Health Disparities (TCHD), Society for the Advancement of Chicanos/Hispanics and Native Americans in Science (SACNAS), Annual Biomedical Research Conference for Minority Students (ABRCMS), STEMConnect, etc. Over the next few weeks, the social media campaign included highlights of each listening session, moderator spotlights, calls to register, a countdown to the sessions, and tips to prepare for each session. LinkedIn Boosts were also utilized to help target healthcare and community outreach initiatives to spread the word. Google Analytics was then used to assess where in the United States people were accessing the registration to determine if any regional areas needed additional promotion. This strategy resulted in 977 registrants from across the United States and territories for the six listening sessions. It was important to ensure that there was equitable distribution based on population across the country to provide as much opportunity as possible for diverse representation within these sessions.

### Session content

During each session, there was an icebreaker, framing activity, guiding questions, and closing questions. The icebreaker was: “We invite you to share: What communities, groups, and/or organizations do you represent?” It was helpful to hear which groups the attendees represented before diving into discussion. The framing activity listed the goals of ACBC: (1) Listen to community voices to learn from their experiences; (2) Capture the AI/ML and health equity needs of individuals/communities to support capacity building; and (3) Gather ideas and direction for the activities of the AIM-AHEAD Consortium through a fair, equitable, and transparent process. Community agreements were also discussed during the framing activity, in order to reiterate that the moderators were there to listen to their needs, concerns, questions, and comments.

There were eight guiding questions to cover throughout each session. To allow as many people as possible the opportunity to speak, a time limit was set for each response. However, attendees who may have been cut short were encouraged to enter their comments in the Slido chat. By evaluating responses from NIH’s previous listening session prior to funding of the A-CC, the planning committees were able to identify topics to include when developing the guiding questions. Furthermore, moderators emphasized that AIM-AHEAD will work to continue this conversation throughout the life of the consortium. Some anticipated topics of discussion included education, research, training, infrastructure, administrative support/human resources, trust, sustainability, and diversity/inclusion. The ACBC Pamphlet that was sent to participants prior to the event included background information on the constructs of AI/ML and health equity. Prompts and clarification were used as needed to guide participants in the discussion. For example, participants requested clarification from the moderators on how to participate in AIM-AHEAD, how to connect with AI/ML experts, and ways to get the community more involved with AIM-AHEAD initiatives. The eight guiding questions were developed to generate discussion around the topics, and are as follows:

1. Tell us about:
  (1.1) Your barriers to using AI/ML;
  (1.2) Ways to overcome some of these barriers;
  (1.3) The resources you might need to address these barriers
2. Tell us about: Your thoughts on the importance of using AI/ML to promote health equity.
3. Tell us about:
  (3.1) Concerns you have in using AI/ML in special or unique populations (ex: rural health, indigenous communities, etc.);
  (3.2) How we can begin to address these concerns and/or biases;
  (3.3) Ways to engage these populations in AI/ML use and put the community first
4. Tell us about:
  (4.1) The best strategies for addressing diversity and inclusion in the field of AI/ML;
  (4.2) What practicing cultural humility should look like when doing this work
5. Tell us about:
  (5.1) Encouraging collaboration among all types of stakeholder groups and promoting multi-disciplinary AI/ML to promote health equity;
  (5.2) Suggestions for building trust
6. Tell us about: What success for AIM-AHEAD would look like to you.
7. Tell us about: Ensuring sustainability to help support communities, organizations, and institutions in the long term.
8. Tell us about: How you would want to participate and engage with the AIM-AHEAD consortium.

The closing questions were intended to summarize all of the discussion in the attendees’ own words and to get specific input from stakeholders about how they felt these items should be actionable moving forward. The closing questions were as follows:

1. What 3–5 words do you feel summarize ALL of the topics discussed?
2. What are the 2 main priorities that AIM-AHEAD should focus on?
3. How can we ensure inclusivity of ideas and insights moving forward? These questions were curated to be open-ended, resulting in an organic generation of themes during a community-based conversation. The questions were developed for community-building discussions rather than for research purposes.

### Data analysis

Data analysts and other A-CC members were present during all of the live sessions. Afterwards, a professional third-party provider transcribed the audio to text files. All transcripts were then de-identified and stored in a secure, password-protected, and restricted-access database. Data analysis procedures included qualitative thematic analysis of voice and chat transcripts [26]. The thematic analysis began with data analysts immersing themselves in the data, to begin looking for insightful quotes and messages within the transcripts. Next, a data coding system was developed. The data analysis team searched for initial patterns and main ideas [26]. Then data codes were linked to overarching themes across all transcripts; this began to illuminate shared ontologies between attendees. This process was iterative and interactive, and as new concepts emerged the data analysis team constructed and reconstructed meaning. The qualitative data analysis was conducted by ELT and AC who both identify as white women and have the roles of evaluators for the AIM-AHEAD Coordinating Center. ELT has expertise in qualitative research methods and both are public health researchers.

Quantitative data analysis procedures included word cloud creation and aggregate counts of stakeholder type representation (e.g., Minority Serving Institutions, community-based organizations, healthcare, etc.). A word cloud (S1 Fig.) was created using quantitative analysis of frequency counts to depict main ideas that arose from the symposium across sessions. The text was pre-processed to exclude moderator introduction/comments or identifying information (names, organizations, institutions, etc.), and combine transcriptions across all sessions.

Frequency counts of phrases were also used, in order to provide context to the text. The word cloud was developed by TA who identifies as a South Asian male from a biomedical informatics, education informatics, and computer science background.

## Results

Out of the 977 people that registered for ACBC, 557 attended. Based on the participants who registered for specific sessions, we defined the attendees as a “community based on a common interest, namely AI/ML.” The number of participants that attended each session are as follows:

- Academic Session: 178 attendees (32% of total)
- Community-Based Session: 73 attendees (13% of total)
- Consortiums Session: 99 attendees (18% of total)
- General Session: 58 attendees (10% of total)
- Healthcare Session: 85 attendees (15% of total)
- Industry Session: 64 attendees (11%)

Key topics that emerged within each area of discussion are listed in Table 1. These topics were then synthesized and six main themes arose, which are listed below. We formulated the six main themes by reading the transcripts, identifying patterns through meaning across all the sessions, and objective word cloud developed from machine readable transcripts. As in any qualitative analysis, subjective experience plays a central role in thematic analysis. The word cloud, developed using https://worditout.com, also helped data analysts visualize main ideas and commonly used words/phrases. After combining the transcripts and comments from all six sessions, removing non-relevant words (e.g., and, the, etc.), and merging like terms (e.g., population/populations, community/communities), we found the words data, AI, communities, people, trust, research, and health equity most prevalent, demonstrating like-minded ideas across all sessions. Results of the ACBC were shared with the AIM-AHEAD Consortium through the AIM-AHEAD Newsletter and social media. However, to retain the privacy of participants, the recordings and transcripts from the sessions were not released.

**Table 1:** Key topics that emerged from ACBC, across sessions, for each guiding question.

Guiding Question	Key Topics from Participant Responses
(1.1) Tell us about your barriers to using AI/ML	• Non-representative data sets • Biased algorithms • Issues with data access, privacy, & security • Lack of infrastructure • Complicated and inconsistent AI/ML language/terminology
(1.2 & 1.3) Tell us about ways to overcome some of these barriers & the resources you might need to address these barriers	• Community outreach and engagement • Meaningful collaboration with stakeholders • Equitable investments in infrastructure, training, and research
(2) Tell us about your thoughts on the importance of using AI/ML to promote health equity	• AI/ML can have positive implications for improving health disparities • Opportunities for AI/ML to predict diagnoses, knowledge, and patterns in health • AI/ML needs to be transparent, translatable, & relatable • Community-oriented AI/ML is essential to developing solutions
(3.1) Tell us about concerns you have in using AI/ML in special or unique populations	• Lack of transparency • Historical and cultural context • Need community buy-in from each different population AIM-AHEAD aims to engage
(3.2 & 3.3) Tell us how we can begin to address these concerns and/or biases & ways to engage these populations in AI/ML use and put the community first	• Connect with existing networks to communicate information about AI/ML to their communities • Build long-term relationships
(4. 1) Tell us about the best strategies for addressing diversity and inclusion in the field of AI/ML	• Encourage interest upstream through promotion and training • Recruitment of diverse individuals • Focus on retention
(4.2) Tell us what practicing cultural humility should look like when doing this work	• Culturally relevant & responsive engagement • Representation matters
(5.1) Tell us about encouraging collaboration among all types of stakeholder groups and promoting multi-disciplinary AI/ML to promote health equity	• Communicate mutual benefits • Build tools together • Create a dedicated space to foster collaboration
(5.2) Tell us about suggestions for building trust	• Address data privacy & security • Tell AI/ML success stories • Ethical AI/ML will lead to increased trust
(6) Tell us what success for AIM-AHEAD would look like to you	• AI/ML best practices & standards • Researcher/workforce diversity • Demonstrate results of AIM-AHEAD initiatives • AI/ML becomes a household word • Funding to under-resourced institutions/organizations
(7) Tell us about ensuring sustainability to help support communities, organizations, and institutions in the long term	• Measurable results are important • Communicating AI/ML real-life examples in everyday life help provide a frame of reference • Continuous stakeholder involvement and empowerment • Utilize existing networks & infrastructure
(8) Tell us how you would want to participate and engage with the AIM-AHEAD consortium	• Co-develop curriculum and lead AI/ML training • Regular communication & outreach from AIM-AHEAD • Develop best-practices & guidelines for AI/ML development and use

### Theme 1. Think global, act local

Several sessions emphasized the importance of using existing networks for facilitating AI/ML activities. The attendees also suggested the involvement of communities on the local level such as barbershops, ministries, community health workers, and citizen scientists. As two session attendees described approaches to reaching community locally:

“In many cultures, there’s a thing, the Griot, right? The Griot, the storyteller, the person that keeps the thing going. The—“train the trainer.” So there’s a lot of ways that we can and have not historically used that whole concept of training the folks who are in there on the ground so that they can then replicate and duplicate and carry on…” (Industry Session).

“I think it’s really important to get in touch with your community health worker network and have these individuals who are very familiar with the area and the people and the boundaries and the cultures to get involved to assist. Because after all these—these individuals, these community health workers, know more about the culture and the aims and can get you there a lot faster. That will in turn help sustain the project.” (Academic Session).

This notion of local networks will help AIM-AHEAD tap into social relationships to reach individuals and organizations at various-levels of AI/ML capacity and sustain activities. Another pattern that was identified in the data relates to global vs. local in terms of the AI/ML models themselves. Participants discussed how generic models would need to be adapted to the community for localized implementation.

“So you can have an AI…that will see different outcomes with different problems that have to be put back into the algorithm in a system that is—I’ll just say, more community oriented. That has a more community focus and [is] understood by the community that you’re actually working with.” (Consortiums Session).

Empowering communities to customize AI/ML tools for their specific local needs will be an important focus for AIM-AHEAD moving forward.

### Theme 2: Diversity, Diversity, Diversity

Diversity, inclusion, and retention were also discussed in all of the sessions. Attendees stated several aspects of the AI/ML field that would benefit greatly from diversity—including (but not limited to) data, workforce, training, and research.

“And the thing we have to be careful about is not homogenizing the group and making sure we don’t try to do that. I remember very explicitly [stakeholder group] said to me, "We can’t lose our cultural identity. We can’t lose the way we look at things just because you want to help all of us." Very explicit. And so we have to be very sensitive about that because we’re trying to help everybody…So we really have to listen and try not to homogenize it because we’ll lose them and we might not even realize we’re losing them. They may be gracious about it and we won’t even know.” (Healthcare Session).

“I’m just speaking for myself. We don’t want people to feel like they were just brought in to be representative, right? We actually want them to be here, right, working with us and trying to figure out how to take the next steps.” (General Session).

Engagement techniques will need to be tailored to each individual stakeholder group’s needs, homogenizing within or across groups should be avoided, and cultural identities should be held to the utmost respect.

### Theme 3. “AI/ML” becomes a household term

ACBC attendees felt there was not enough information readily available that clearly articulated how AI/ML can be used in everyday life. ACBC attendees communicated that AI/ML needs to be demystified, with hopes that “AI/ML” would become a household term. The attendees also pointed out the need for ongoing points of engagement through future listening sessions, social media, and other tailored community-centric approaches.

“Let’s engage our neighbors. Let’s have them part of the conversation because again, this is frightening to them, artificial intelligence. Let’s get that out in the open and demystify this. That means that we have to demystify and be humble and talk plain language to the very communities that are suffering.” (Academic Session).

“Sci-fi can make AI seem deterministic or flawed or whatever. And if that’s the way that most people perceive AI or, or consume information about AI, it doesn’t really help our case. So you have to sort of humanize the process of creating algorithms and models and saying, okay, they’re it… They’re influx, they can change, they can be improved and we’re not always listening to like our robot overlords for, for all advice.” (General Session).

There should be standards for language and terminology in the field of AI/ML to accommodate collaboration across disciplines. Providing context about how algorithms can be improved allows for a greater understanding and reduces fear of the unknown about the topic.

### Theme 4. Barriers exist, but can be overcome

ACBC attendees listed barriers they have personally faced. These barriers included general lack of awareness, limited access to data/infrastructure/training, and insufficient support systems around AI/ML. However, the attendees also provided potential solutions to overcome these barriers. Some areas for improvement included: collaboration, equitable distribution of resources, providing education and training, creating a standardized repository to foster data sharing across disciplines, addressing bias in data and algorithms, acknowledging historical and social context, and applying ethical considerations.

“What we are seeing right now is, the biggest barrier, what we are seeing is access and opportunity. And how we can overcome this is really exposure and training so people are aware of really what artificial intelligence and machine learning is. And once we’re able to get to that point, then they can trust certain systems.” (Community-Based Session).

“What I think the biggest challenge I’ve had… barrier to using machine learning and AI is having adequate representative data sets to test and train your algorithms on. If there was a way that we could work with some of the organizations like NIH to create these test and training data sets for medical knowledge that were de-identified, that would really help with the development of these technologies.” (Academic Session).

“I think a real impact will be made when we can get people that are non-STEM background to not look at AI and ML as “that other science,” but see it as something that they can do themselves and then they will apply it to their appropriate disciplines in ways that a STEM person probably would never think of.” (Academic Session).

By gaining greater insight into the needs of stakeholders, AIM-AHEAD is able to better gear programs and interventions to target population interests. Additionally, having those groups discuss proposed solutions opens the dialogue for shared decision making and creates a sense of comradery for overcoming barriers together.

### Theme 5. Open science platform for health equity

Discussions also centered around making AI/ML more transparent, accessible, and explainable—dismantling the “black box.” In using AI/ML to promote health equity, there needs to be a balance of access to data while protecting privacy. Therefore, an open science platform could be used. Aspects of privacy and security should be emphasized when creating these tools, especially when incorporating EHR and SDoH data into a dedicated space for collaboration. Additionally, a platform such as this could be beneficial in disseminating information back to the community from where it came.

“I always think about what are going to be the financial impacts, particularly on pharmaceutical companies and the like. I think about Henrietta Lacks, the drugs and the profits that were made from that. I kind of think about, well, there’s going to be profit even in healthcare. How is that somehow shared in some way, shape, or form with those who are contributing the data.” (Academic Session).

“So it’s leveraging AI/ML to promote health equity…it’s understanding what data are you using, how you’re using it, what algorithms you’re using, how often you look at them, what decisions you’re making and being very open and honest and transparent about it, and actually testing yourself.” (Healthcare Session).

"One of the other areas that I’m thinking about because I’m coming from the medical community is that our medical platforms don’t always communicate with each other. So if you are using one electronic record, someone else may be using a different format and those systems do not communicate. If you’re thinking of transferring patient information and utilizing that, you need to have it capable of communicating across various platforms. Otherwise it becomes frustrating." (Healthcare Session).

Part of what is needed to make AI/ML an effective tool to address health disparities is a multi-disciplinary approach. However, collaboration amongst different groups will require platforms to share data, AI/ML approaches, and results. An open science platform could provide a space to work together in a transparent and equitable way.

### Theme 6. Paving pathways to success

Attendees listed several ideas that they felt would contribute to the overall success of AIM-AHEAD. AIM-AHEAD should have stakeholders involved in every step of the process of building the consortium, which will in turn lead to buy-in from communities. Capacity building through education, training, mentoring, and supporting a diverse pipeline of AI/ML researchers/workforce were also considered markers of success. Many attendees were interested in engaging with the AIM-AHEAD Consortium after ACBC.

“I think success in my estimation would be you have readily accessible, interoperable, high quality system that it doesn’t matter whether you’re in Dallas or California or New York or Mississippi, the level and quality of the services that you’re providing through AI/ML is of the same level. And it doesn’t matter whether you have $2 billion or you have $2, you still have the same access and use of that information and your health outcomes are going to be high quality, mortality rates going to be down.” (Healthcare Session).

Participants shared many ways in which they felt AIM-AHEAD could improve reach, collaborate with existing data science programs, and overcome current challenges in the field of AI/ML and health equity. Engaging stakeholders in refining visionary ideals of success and setting program goals also increases sustainability of the initiative.

## Discussion

For social, behavioral, and biomedical fields to benefit from advanced technologies such as artificial intelligence and machine learning, it is important to first understand the needs of those receiving and providing care before moving forward with any advancement or change [4,5,6]. This paper summarizes the findings from the six community listening sessions conducted in February 2022 on the use of AI/ML to achieve health equity and expand workforce diversity in AI/ML. The listening sessions were attended by diverse members of the community across academia, industry, nonprofit organizations, and local communities. These listening sessions crystalized and reinforced the need for continuous community engagement, input, and feedback to inform the current implementation and future planning of A-CC activities. This discussion was guided by the two overarching goals of the A-CC: “promoting health equity” and “expanding workforce diversity” in AI/ML. The nation-wide listening sessions offered a tangible opportunity to voice concerns of under-represented/under-resourced communities. Each session offered a unique perspective and experience, and acknowledged the need for significant contribution from all sectors to tackle the problem of health disparities and achieve health equity. Attendees across all listening sessions acknowledged the significant potential of AI/ML to promote health equity. However, thematic analysis revealed six major themes. These themes emphasized the need to “think globally and act locally,” promote “diversity” in the entire AI/ML ecosystem, “demystify/communicate” to increase AI/ML awareness and knowledge, need for activities to “overcome barriers,” and to promote “open science.” A final theme highlighted the need to engage in a deliberate process and “clearly define what success looks like.”

The thematic analysis also revealed recommendations that emerged from the listening sessions. These included promoting awareness of AI/ML through podcasts, webinars, inclusion of diverse stakeholders, clarification of the goals of A-CC, achieving equity in entire ecosystem of A/ML such as: data quality, identifying and using diverse sources of data, training algorithms to recognize the diversity, and developing/deploying AI/ML models through the lens of health equity. The listening sessions also reinforced the need for integrating cultural humility into all programs and activities of the A-CC. The focus sessions confirmed some of the existing barriers to cross-collaboration among health sciences such as bridging the gap in cross-domain knowledge, lack of capacity in under-represented institutions. Moreover, new knowledge emerged because existing knowledge typically comes from white papers and reports from non-academic work such as Harvard Business Review. Scholarly literature in this area is limited and studies have focused on patients and not the larger public from diverse communities [27]. Thus, new insights were gained that can guide future planning. It has to be noted that the purpose of these group sessions was to inform practice rather than research.

### Future planning

Key messages from the listening sections will guide the A-CC in how to engage with organizations/institutions/communities, develop use cases, and communicate AI/ML terminology and success stories. The shared perspectives and experiences of the community members have also guided the implementation of first year activities at an accelerated pace and planning of future programming/initiatives. For example, efforts were directed toward a smaller, more targeted scale in order to dive deeper into ideas for carrying out tailored approaches for local communities. In response to the call for increasing awareness on AI/ML, a two-day free virtual symposium on AI/ML and health equity was held titled “AI for Health Equity Symposium” (AIHES) (https://aim-ahead.net/convention/p/aihes). As part of AIHES, to expand capacity in health equity and AI/ML, free training opportunities were provided in July 2022 through a month-long series of workshops covering a wide range of health equity and AI/ML topics.

Diversity in capacity-building efforts and projects is also a main focus of the A-CC. As such, AIM-AHEAD started a Research Fellowship (https://aim-ahead.net/ResearchFellows) and a Leadership Fellowship (https://aim-ahead.net/fellows/LeadershipFellows) with a significant focus on diversity and inclusion. Applicants from diverse backgrounds were encouraged to apply, and the first cohorts for both fellowships proudly represent a diverse pool of individuals.

In terms of engaging a diversity of disciplines in this field, the AIM-AHEAD Data Science Training Core plans to create asynchronous training courses for both STEM and non-STEM, novices and experts, students and researchers through AIM-AHEAD Connect. Furthermore, through publicly available webinars, AIM-AHEAD has been able to virtually engage a diverse range of stakeholders. A strong feature of the AIM-AHEAD leadership structure is the inclusion of regional hubs. These hubs are able to recruit stakeholders to participate in these activities and listen to local community needs related to AI/ML and health equity. After the ACBC, many of the hubs hosted their own, more intimate listening sessions and have created in-person and online spaces to continue connecting with the communities.

With the goal of promoting health equity through research, a series of pilot projects have been funded by AIM-AHEAD. These pilots also encourage diverse researchers to apply, with a focus on community and AI/ML innovation. In the future, AIM-AHEAD plans to continue to engage stakeholders in a fair and equitable way to listen to their needs on an ongoing basis. These recommendations are extremely important to ensure true partnerships are created with the stakeholders who have opportunities to be involved in decision-making processes. Given attendees emphasized addressing bias in data and algorithms during ACBC, the pursuit of ethical AI will be a key part of AIM-AHEAD’s second year activities. More information about A-CC activities following ACBC will be shared in subsequent manuscripts.

### Strengths & limitations

Strengths of ACBC include a nationwide reach, involvement of regional hubs in promoting the event, enthusiasm of A-CC members in active participation throughout every step of planning and executing the event, and the ability to plan ACBC in a short time frame. AIM-AHEAD was able to reach a diverse audience to attend the sessions, and in the future it would be desirable to have more widespread participation from other priority communities (e.g., rural communities, small start-ups, under-resourced minority serving institutions, public health organizations, etc.). Some limitations of information gathered in the listening sessions include selection bias and social desirability bias [28]. First, selection bias could have occurred since ACBC was marketed based on existing partnerships. Second, social desirability bias could have come into play for attendees who did not decide to remain anonymous (i.e., through their Zoom Display Name and/or by having their camera on). Third, there are limitations to the consortium model itself. Morrison and colleagues (2020) describe some challenges of conventional research consortia to be issues of governance, stakeholder integration, transparency, and future planning. It will be important for the AIM-AHEAD Consortium to work closely with funders to ensure that priorities closely align, and contracts are designed to clearly outline governance processes that are able to sustain themselves even after the project ends [29]. Additionally, data were analyzed by the authors who have backgrounds that do not necessarily represent the desired diversity of the consortium. This positionality of the authors is disclosed.

## Conclusion

AI/ML still remains a mystery to many, especially when it is used in healthcare settings, which can lead to mistrust. Equitable partnerships are needed to overcome this barrier. AIM-AHEAD is developing and growing a national consortium of patients, researchers, other individuals, organizations, institutions, and communities to address challenges in AI/ML and health equity. Potential target audiences to collaborate with when developing AI/ML tools for health equity will likely depend on the specific context of local communities. Therefore, it will be important for AIM-AHEAD to continue to draw upon the strengths of the local hubs to reach key stakeholders from different disciplines, demographic areas, and regions. The aim of ACBC was to connect with AIM-AHEAD stakeholders/partners and continue the conversation NIH first initiated prior to funding the program to assess data, infrastructure, and training needs. The listening sessions indeed provided rich insight into the AI/ML needs around these topics. AIM-AHEAD was able to contribute to immediate outcomes and share these reflections with the A-CC, communities who participated, and NIH networks. The findings of ACBC are relevant to a wider audience of those in the field of AI/ML, data science consortiums, and healthcare as they shed light on areas to make implementation of these tools more sustainable and resilient. AIM-AHEAD will use these findings to contribute to practice both internally and externally. Furthermore, it is important to create a space where stakeholders from various disciplines can share concerns, discuss ideas, and foster innovation. As such, AIM-AHEAD will continue to engage with stakeholders through a variety of mediums in order to promote an open dialogue around AI/ML and health equity.

## Supporting information

S1 Fig Word cloud depicting common themes across ACBC sessions. This word cloud was derived from all six listening sessions and depicts the most prominent themes across all sessions. The largest word, “data” in blue, reveals that this was the most common word spoken, followed by “AI,” “communities,” “people,” “need,” and so on as the words decrease in size. http://bit.ly/3V5jqog
(PNG)
