# Correction: Community perspectives on AI/ML and health equity: AIM-AHEAD nationwide stakeholder listening sessions

**DOI:** 10.1371/journal.pdig.0001177

**Published:** 2026-01-02

**Authors:** Jamboor K. Vishwanatha, Allison Christian, Usha Sambamoorthi, Erika L. Thompson, Katie Stinson, Toufeeq Ahmed Syed, AIM-AHEAD Consortium

AIM-AHEAD Consortium is not included in the author byline. AIM-AHEAD Consortium should be listed as the seventh author. The contributions of this author are as follows: Contributed in the development or design of methodology.

The fourth author, Erika L. Thompson, is incorrectly noted as the corresponding author. The correct corresponding author is Toufeeq Ahmed Syed. Toufeeq Ahmed Syed’s email address is: toufeeq.ahmed@vumc.org
