# Supplementary figures and images for: Community perspectives on AI/ML and health equity: AIM-AHEAD nationwide stakeholder listening sessions
Source: PLOS Digit Health. 2023 Jun 30;2(6):e0000288. doi: 10.1371/journal.pdig.0000288 (PMC10313007; doi:10.1371/journal.pdig.0000288)

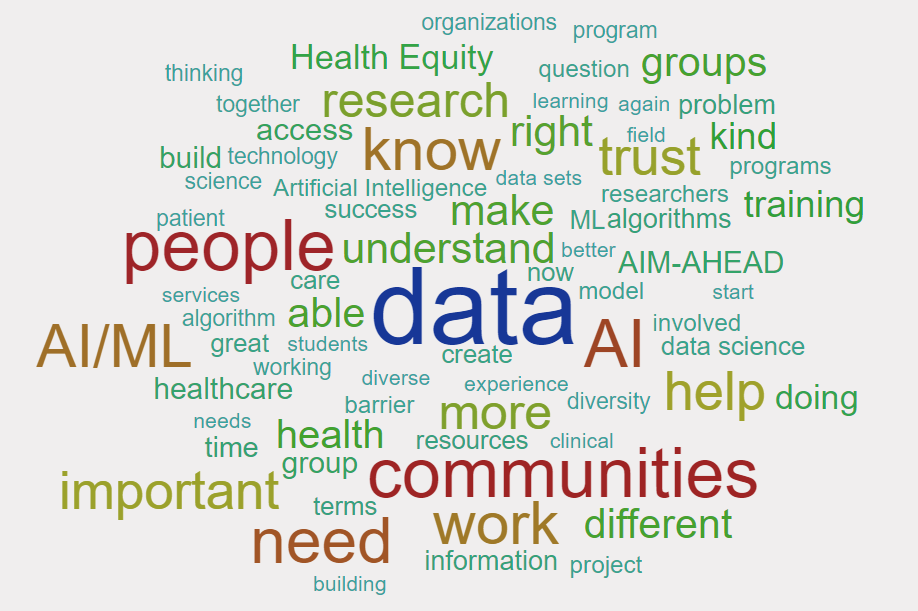

Supplement: S1 Fig — This word cloud was derived from all six listening sessions and depicts the most prominent themes across all sessions. The largest word, “data” in blue, reveals that this was the most common word spoken, followed by “AI,” “communities,” “people,” “need,” and so on as the words decrease in size. http://bit.ly/3V5jqog (PNG) [file pdig.0000288.s001.png]
